# Clinical Evaluation of Corneal Endothelial Parameters following Laser Refractive Surgery in Myopic Eyes: A Review

**DOI:** 10.3390/jcm13061665

**Published:** 2024-03-14

**Authors:** Maciej Juda, Maciej Bedliński, Anna Maria Roszkowska, Joanna Wierzbowska

**Affiliations:** 1 Department of Ophthalmology, Military Institute of Medicine—National Research Institute in Warsaw, 04-141 Warsaw, Poland; mjuda@wim.mil.pl (M.J.); mbedlinski@wim.mil.pl (M.B.); 2 Ophthalmology Clinic, Department of Biomorphological Sciences, University of Messina, 98124 Messina, Italy; aroszkowska@unime.it; 3 Ophthalmology Section, Faculty of Medicine and Health Sciences, Andrzej Frycz Modrzewski Kraków University, 30-705 Kraków, Poland; 4 Optegra Eye Clinic, 02-366 Warsaw, Poland

**Keywords:** laser refractive surgery, laser in situ keratomileusis, photorefractive keratectomy, myopia, corneal endothelium

## Abstract

**Background:** The aim of this review was to investigate the influence of various laser refractive surgery methods on the corneal endothelium in myopic patients. The role of the corneal endothelium in laser refractive surgery (LRS) is currently being addressed in the assessment of postoperative corneal edema risk. **Methods**: Changes in corneal endothelial cell density and morphology after LRS were evaluated based on a systematic review of current studies. The results of a literature search in the PubMed, Science Direct, Google Scholar, and the Web of Science databases, as well as a manual search, were selected for the final review according to the PRISMA 2020 flow diagram. **Results**: We included 24 prospective clinical trials in the review: surface ablation (twelve), LASIK and FemtoLASIK (two), femtosecond lenticule extraction (two), and comparable studies (eight). Endothelial cell density was determined by specular or in vivo confocal microscopy. In most studies, no statistically significant differences were found between preoperative and postoperative endothelial parameters. In nine studies, the changes were statistically significant, but no vision-threatening complications occurred, and no serious corneal complications developed in any eyes during the follow-up period. **Conclusions**: Based on collected data, laser keratorefractive surgery appears not to exert a significant effect on the corneal endothelium.

## 1. Introduction

Refractive errors are the main global cause of reversible visual deterioration. They may be corrected via various methods, including laser corneal refractive procedures, which are the most frequent ocular surgeries performed worldwide [1,2]. Myopia (nearsightedness) is the most common type of refractive error [2], and its increasing prevalence throughout the world has become a major public health problem. It is estimated that billions of people will be affected by this condition by 2050 [3]. The correction of myopia and myopic astigmatism accounts for over 80% of laser refractive procedures [4]. There is growing evidence that LRS offers not only a favorable safety profile, but also excellent visual results, enabling complete independence from glasses or contact lenses in the majority of cases, which significantly improves the patients’ quality of life [5,6].

Various techniques of LRS are currently available. Surface techniques, such as photorefractive keratectomy (PRK) or laser-assisted subepithelial keratectomy (LASEK), as well as stromal techniques including laser in situ keratomileusis (LASIK) and small incision lenticule extraction (SMILE), are widely used all over the world.

The cornea consists of five layers: the epithelium, the Bowman’s layer, the stroma, Descemet’s membrane, and the endothelium. Each of these layers plays a key role in maintaining corneal transparency and optimal visual function. The endothelium, a single layer of hexagonal endothelial cells covering the inner surface of the cornea, is responsible for regulating stromal hydration by acting as a passive barrier to the uptake of fluid from the aqueous humor and actively pumping fluid out of the cornea. The normal human cornea experiences an annual reduction rate of 0.6% in central corneal endothelial cell density (ECD) [7]. At birth, the ECD is approximately 3000–4000 cells/mm^2^, decreasing to 2500 cells/mm^2^ in late adulthood [8]. Research on normal and eye bank corneas has indicated that the paracentral and peripheral zones have endothelial cell densities 5% and 10% higher than the central cornea, respectively. Unlike the corneal epithelium, endothelial cells do not undergo mitosis in vivo. Instead, they rely on migration to recover from injury and cell death. In case of a localized cell loss in the central area, adjacent endothelial cells move toward the center. Compensatory mechanisms involve the enlargement of healthy cells (polymegathism) to replace the lost cells on the corneal back surface, resulting in decreased hexagonality (pleomorphism) [9]. Endothelial decompensation, typically occurring when cell density falls below 500 cells/mm^2^, leads to overhydration, causing corneal edema with increased stromal thickness, bullous keratopathy, and scarring, ultimately resulting in irreversible visual acuity loss [10].

Nowadays, in vivo images of the human corneal endothelium may be reliably obtained through specular [11] and in vivo confocal microscopy [12], which enable the calculation of endothelial cell density (ECD) and two morphological indices: a coefficient of cell area variation (CV), which is a measure of the homogeneity of the endothelial cell population, and the percentage of hexagonal cells (HEX).

Various mechanisms may have a potential impact on the corneal endothelium during laser refractive procedures. Risks generated by an excimer laser include mechanical trauma from shock waves, oxidation, and thermal effects from UV light [13,14] that may harm fragile endothelial cells. Accumulated energy delivered in procedures involving both femtosecond and excimer lasers (FemtoLASIK) may contribute to even greater corneal endothelial cell damage [15,16]. LVC procedures may contribute to generating cytokines and other inflammatory factors leading to subclinical diffuse lamellar inflammation (DLK), inflammation, or flares in the anterior chamber, which may also affect the endothelium [17]. In general, the depth of ablation needs to increase with greater degrees of myopia. Although the threshold of at least 250 µm of postoperative stroma thickness [18] is recommended, too-deep ablation along with an excessively thin residual bed may pose a further risk to the corneal endothelium. Moreover, mitomycin C (MMC), a drug which is commonly used during surface ablations to prevent postoperative haze, may be detected in the aqueous humor postoperatively and may also raise concerns over its possible endothelial toxicity [19]. Some authors have reported cases of corneal decompensation in eyes with guttae or Fuchs endothelial dystrophy after LASIK [20,21].

Due to the multitude of possible treatment options, choosing the safest and most effective method for laser vision correction seems to be a crucial challenge for refractive surgeons nowadays. The purpose of this review was to analyze the available literature and provide up-to-date information on whether superficial laser refractive procedures as well as LASIK, FemtoLASIK, and femtosecond lenticule extraction may have long-term adverse effects on the corneal endothelium.

## 2. Materials and Methods

This study was designed according to the PRISMA 2020 flowchart for new systematic reviews, which includes database and registry searches.

The results of a literature search on corneal endothelial changes after laser refractive surgery were included in the meta-analysis. The following databases were analyzed: PubMed, Scopus, Google Scholar, and the Web of Science. Studies omitted by automatic filters and found by manual searching were also taken into account. The following terms and phrases were used in the database search strategy: (“Endothelium, Corneal” OR “corneal endothelial loss” OR “corneal decompensation” OR “corneal endothelium” OR “corneal guttae” OR “Fuchs’ endothelial corneal dystrophy”) AND (“Corneal Surgery, Laser” OR “refractive surgery” OR “laser in situ keratomileusis” OR “femtosecond laser” OR “femtosecond laser assisted in situ keratomileusis” OR “laser assisted subepithelial keratomileusis” OR “transepithelial photorefractive keratectomy” OR “small incision lenticule extraction” OR “SMILE” OR “photorefractive keratectomy” OR “laser subepithelial keratomileusis” OR “epipolis-laser in situ keratomileusis” OR “transepithelial” OR “photorefractive keratectomy” OR “epi-Bowman keratectomy” OR “sub-Bowman keratomileusis”) NOT keratoplasty NOT cataract.

The publications were reviewed and checked for suitability by three of the authors: M.J., M.B., and J.W. The review of the abstracts and, later, the full texts of the articles led to the final selection of the literature. Only papers that met the inclusion criteria and not the exclusion criteria were considered in the final selection of articles. The following inclusion criteria were applied: (i) prospective studies published in English; (ii) follow-up of at least 6 months; (iii) refractive surgery method used: PRK, transepithelial photorefractive keratectomy (Trans-PRK), LASEK, LASIK, femtosecond laser-assisted in situ keratomileusis (FemtoLASIK), ReLEx SMILE; (iv) myopic population; (v) corneal endothelial parameters (one or more): mean endothelial cell density (ECD), coefficient of variation (CV, polymegathism index), percentage of hexagonal cells (HEX, polymorphism index); (vi) in vivo confocal microscopy (IVCM); and (vii) specular microscopy (SM). The exclusion criteria were: (i) animal studies, retrospective studies, experimental studies, reviews, single-case reports, letters to the editors, editorials, conference abstracts, meta-analyses, main text in a language other than English.

**Figure 1:**
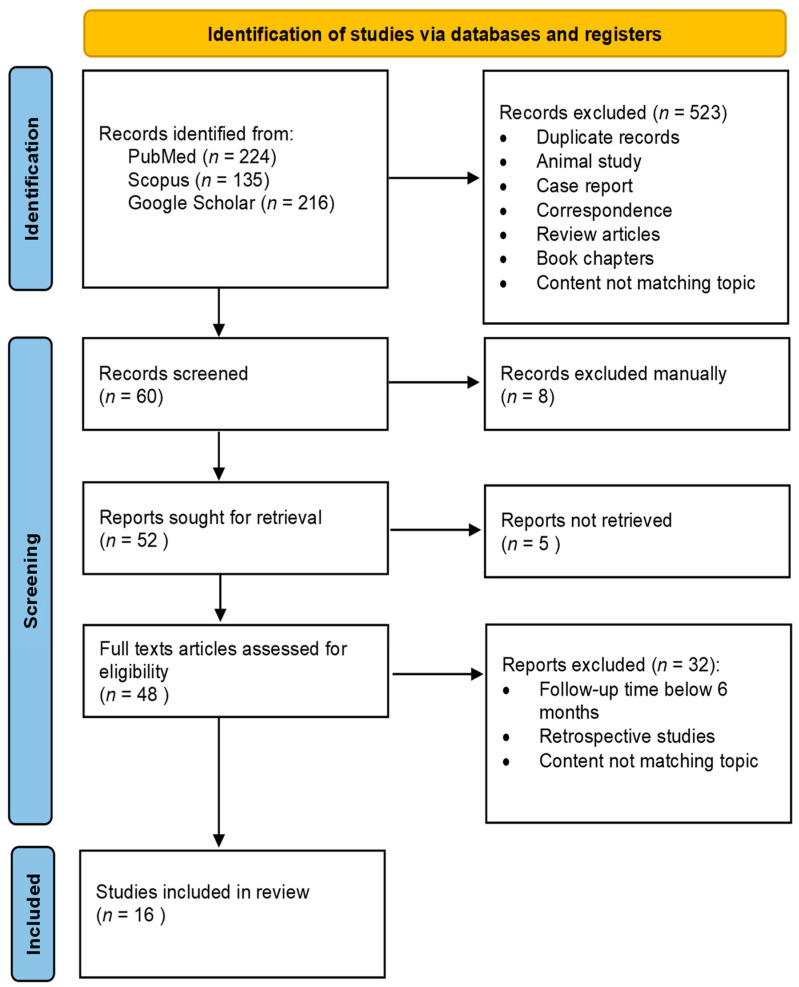
Prisma 2020 flow diagram for the identification of studies.

The process of literature selection for the systematic review is shown in the PRISMA 2020 flowchart (Figure 1). After the removal of duplicates, the titles and abstracts of all articles were checked for suitability by two independent authors (M.J. and M.B.). Questionable articles were discussed in detail by the authors, and the senior investigator (J.W.) made the final decision. Finally, a total of 24 studies were eligible for the review. Clinical data on the study size, age, preoperative spherical equivalent (SE), treatment method, ablation depth (AD), length of follow-up (FU), pre- and postoperative ECD, CV, and HEX were reviewed.

## 3. Results

Through the literature search and screening, 24 articles on the clinical evaluation of corneal endothelial parameters following laser refractive surgery in myopic eyes, published between 1993 and 2000, were included in this review. Eleven studies focused on PRK only, one study focused on LASEK [22], one focused on Trans-PRK [23], two focused on LASIK or FemtoLASIK [24,25], and two analyzed SMILE [26,27]. Seven remaining studies were comparative trials, three studies focused on PRK and LASIK [28,29,30], one focused on PRK and LASEK [31], one focused on PRK and Epi-LASIK [32], one focused on LASIK and FemtoLASIK [33], and one analyzed FemtoLASIK and SMILE [34]. Five studies explored PRK or LASEK with an intraoperative 0.02% MMC application [22,23,32,35,36].

The review encompassed data from 1364 eyes belonging to 1120 patients at an average age of 25.46 years (ranging from 18 to 60). Nine articles included patients with a spherical equivalent (SE) below −6.00 diopters (D), while 15 articles encompassed SE ranges exceeding −6.00 D. The authors of 20 articles used SM to measure ECD, CV, and HEX. In the remaining four articles, the authors used IVCM to investigate those parameters.

Various methods were employed to evaluate endothelial parameters through the use of SM and IVCM. Specifically for SM, automated computerized analysis was used in 15 studies, the frame method was used in two studies, the comparison method was used in one study, and two authors did not specify the calculation method. As regards IVCM, computer analysis was used in two articles, the frame method in one article, and the center method in one article. In four papers, endothelial measurements were taken in both the central and peripheral zones, while 20 articles focused exclusively on the central endothelium. In six studies, SM or IVCM measurements were performed by one technician. In the remaining 20 studies, the information about the personnel performing endothelial measurements is unavailable.

In 22 out of 24 studies, an excimer laser was used. The repetition rate ranged from 5 Hz to 500 Hz and the laser energy ranged from 90 to 500 mJ/cm^2^. Ten studies provided no information regarding either the energy or the repetition rate of the excimer laser.

The average optical zone (OZ) was 5.65 mm, varying from 4.20 to 7.0 mm. The OZ ranged from 4.0 to 5.0 mm in six studies, from 5.1 to 6.0 mm in five studies, and from 6.1 to 7.0 mm in two studies. The authors of three studies used various OZs. No information on the OZ diameter was included in six studies. The minimum follow-up duration was 6 months, while the maximum reached 108 months. In two studies, only a femtosecond laser was applied [26,27].

In most articles, no statistically significant differences were found between preoperative and postoperative endothelial parameters. In ten articles, the changes were statistically significant. The authors of three studies identified statistically significant changes in all three analyzed endothelial parameters following laser refractive surgery (Trocmé et al. [37], Patel et al. [29], Shaaban et al. [34]). Two of those investigations revealed a decrease in both ECD and CV, along with a simultaneous increase in HEX (Trocmé et al. [37], Patel et al. [29]). In contrast, one study reported a decrease in both ECD and HEX, while CV increased (Shaaban et al. [34]). Some other authors observed either a significant increase in ECD (Muñoz et al. [25]) or a significant decrease in ECD (Nassiri et al. [35], Pallikaris et al., 1994 [28]). Significant alterations in CV were observed in three other papers, whose authors indicated either a decrease in CV (Adib-Moghaddam et al. [23]) or an increase in CV after the laser procedure (Gharaee et al. [36]). In one of those studies, a significant increase in HEX was also noted (Carones et al. [38]).

### 3.1. Surface Ablation

Of the 18 included articles, 13 studies analyzed corneal endothelial changes following a single surface ablation (SA) procedure and five studies also compared postoperative endothelial parameters between PRK and LASEK groups [31], PRK and Epi-LASIK groups [32], or PRK and LASIK groups [28,29,30]. Characteristics and results of surface ablation studies are presented in Table 1.

No significant differences in ECD, CV, and HEX were reported between the preoperative and postoperative values in eight out of eighteen analyzed studies [22,30,31,39,40,41,42,43]. A significant decrease in ECD following PRK was noted by three authors (Trocmé et al. [37], Nassiri et al. [35], Pallikaris et al., 1994 [28]). In the study by Trocme et al. [37], the mean ECD for the peripheral zone was significantly decreased, whereas the mean ECD for the central zone did not differ from the preoperative value at 12 months after PRK. In turn, Pallikaris et al. (1999) [44] observed a significant increase of 3.3% in the mean ECD in the central zone at 12 months after PRK, whereas no change was noted in ECD in the peripheral zone.

A significant decrease was noted in CV following SA procedures in four studies (Carones et al. [38], Mardelli et al. [45], Trocmé et al. [37], Patel et al. [29]). In turn, CV improved after PRK in two studies (Gharaee et al. [36], Adid-Moghaddam et al. [23]). A statistically significant increase in cell hexagonality following SA procedures was noted by three authors (Carones et al. [38], Trocmé et al. [37], Patel et al. [29]).

**Table 1:** Characteristics and results of surface ablation studies.

Study	Study Size		Age (Mean, SD, Range) (Years)	Correction Method	Excimer Laser Firing Frequency (Hz)	Exam Method	Follow-Up Time (Mean, SD, Range) (Months)	Ablation Depth (Mean, SD, Range) (µm)	SE (Mean, SD) (Diopters)	ECD (Mean, SD)		CV (Mean, SD)		HEX (Mean, SD)	
	Patients	Eyes							Pre-Op	Pre-Op	Post-Op	Pre-Op	Post-Op	Pre-Op	Post-Op
Amano S. et al., 1993 [39]	20	26	33, N/S, (22–57)	PRK	N/S	SM	12, N/S, N/S	38, N/S, (19–50)	−5.80, N/S, (−2.75–−10.50)	3221 ± 216	3177 ± 185	0.24 ± 0.09	0.22 ± 0.05	N/S	N/S
Carones F. et al., 1994 [38]	61	76	30, N/S, (20–49)	PRK	10	SM	12, N/S, N/S	72, N/S, (24–113)	−6.60, N/S, (−1.75–−13.50)	2657 ± 298	2656 ± 289	30.27 ± 5.99	26.35 ± 5.29 *	63.8 ± 9.9	67.1 ± 9.1 *
Pallikaris I.G. et al., 1994 [28]	20	10	N/S, N/S, (22–46)	PRK	20	SM	12, N/S, N/S	N/S	−13.65 ± 1.21 (−10.75–−23.12)	2800 ± 218	2690 ± 239 *	N/S	N/S	N/S	N/S
Mardelli P.G. et al., 1995 [45]	32	35	39, N/S, (22–57)	PRK	5	SM	38, N/S, (12–54)	N/S	N/S	2950 ± 329	2907 ± 337	0.29 ± 0.01	0.28 ± 0.05 *	65.8 ± 5.6	64.2 ± 5.6 *
Trocmé S.D. et al., 1996 [37]	14	28	N/S	PRK	10	SM	12, N/S, N/S	N/S	N/S, N/S, (−2.00–−6.00)	A: 2889 ± 283	2988 ± 292	A: 0.30 ± 0.05	0.28 ± 0.05 *	A: 58.6 ± 10.9	64.1 ± 11.0 *
										B: 3086 ± 320	2966 ± 332	B: 0.31 ± 0.05	0.27 ± 0.05 *	B: 57.1 ± 9.2	63.5 ± 9.7 *
										C: 3184 ± 385	2963 ± 296 *	C: 0.32 ± 0.06	0.27 ± 0.04 *	C: 60.1 ± 8.6	63.7 ± 7.6
Spadea L. et al., 1996 [40]	50	50	34 ± 10 (20–60)	PRK	10	SM	11 ± 6 (6–34)	67 ± 20 (31–111)	−7.80 ± 3.70 (−2.50–−17.00)	2674 ± 398	2578 ± 402	N/S	N/S	N/S	N/S
Pallikaris I.G. et al., 1996 [41]	42	42	29 ± 6, N/S, N/S	PRK	N/S	SM	6, N/S, N/S	N/S	−4.13 ± 1.24 (−1.62–−6.12)	3115 ± 322	3220 ± 333	N/S	N/S	N/S	N/S
Cennamo G. et al., 1997 [42]	18	36	30, N/S, (19–57)	PRK	20	SM	12, N/S, N/S	91 ± 12(62–116)	−10.30 ± 1.40, N/S	2818 ± 337	2894 ± 301	79.00 ± 2.30	81.00 ± 5.00	N/S	N/S
Frueh B.E. et al., 1998 [43]	12	18	35 ± 12, N/S	PRK	13	IVCM	17 ± 8 (12–24)	(35–100)	−5,30 ± 2.10, N/S	2678 ± 311	2658 ± 256	N/S	N/S	N/S	N/S
Pallikaris I.G. et al., 1999 [44]	102	102	31 ± 7 (19–52)	PRK	32	SM	12, N/S, N/S	N/S	−4.22 ± 1.22 (−1.50–−6.25)	2593 ± 377	2678 ± 358	N/S	N/S	N/S	N/S
Diakonis V.F. et al., 2007 [32]	15	15	N/S	PRK MMC(+)	400	IVCM	12, N/S, N/S	50 ± 12, N/S	−3.32 ± 0.48 (−2.00–−4.75)	2757 ± 117	2721 ± 113	N/S	N/S	N/S	N/S
		15	N/S	Epi-LASIK				53 ± 10, N/S	−3.54 ± 0.65 (−2.75–−5.00)	2769 ± 158	2760 ± 102	N/S	N/S	N/S	N/S
Zhao L. et al., 2008 [22]	89	174	28 ± 6 (18–45)	LASEK MMC(+)	120	SM	6, N/S, N/S	134 ± 35 (52–224)	−7.68 ± 2.74 (−1.95–−14.95)	2755 ± 373	2770 ± 399	31.45 ± 8.26	32.55 ± 9.07	66.0 ± 25.8	70.7 ± 24.3
Nassiri N. et al., 2008 [35]	48	76	29 ± 7 (18–47)	PRK MMC(+)	N/S	SM	6, N/S, N/S	81 ± 18, N/S	−4.19 ± 1.27 (N/S)	2740 ± 361	2682 ± 385 *	N/S	N/S	N/S	N/S
	33	86		PRK MMC(−)				43 ± 12, N/S	−2.14 ± 0.81 (N/S)	2744 ± 333	2752 ± 316 *	N/S	N/S	N/S	N/S
Patel S. et al., 2009 [29]	16	9	39 ± 6 (31–44)	PRK	N/S	SM	108, N/S, N/S	31 ± 19, N/S	−3.50 ± 1.70 (−1.25–−5.75)	2641 ± 340	2559 ± 423	0.36 ± 0.03	0.32 ± 0.04 *	51.0 ± 5.0	58.0 ± 5.0 *
Amoozadeh J. et al., 2009 [30]	12	24	28 ± 6 (20–35)	PRK	N/S	IVCM	6, N/S, N/S	62 ± 15 (38–69)	−2.85 ± 0.99 (−1.00–−4.00)	2983 ± 293	3025 ± 404	N/S	N/S	51.3 ± 11.0	53.3 ± 10.2
Sia et al., 2014 [31]	167	334	34 ± 8 (21–57)	PRK MMC(+)	N/S	SM	12, N/S, N/S	83 ±16 (56–129)	−5.99 ± 1.40 (−3.88–−9.38)	2914 ± 352	2859 ± 298	N/S	N/S	N/S	N/S
				LASEK				83 ± 16 (53–114)		2787 ± 329	2860 ±407	N/S	N/S	N/S	N/S
				PRK				84 ± 16 (56–120)		2870 ±375	2828 ±345	N/S	N/S	N/S	N/S
Gharaee H. et al., 2018 [36]	48	96	27 ± 5 (18–34)	PRK MMC(+)	100	SM	6, N/S, N/S	N/S	−3.57 ± 0.18 (−0.50–−6.75)	2800 ± 37	2764 ± 38	0.37 ± 0.00	0.40 ± 0.10 *	39.7 ± 1.3	36.9 ± 1.3
Adib-Moghaddam S. et al., 2018 [23]	71	142	28 ± 6 (22–45)	Trans-PRK MMC(+)	500	SM	12, N/S, N/S	122 ± 22, N/S	−3.20 ± 1.23, N/S	2864 ± 295	N/S	30.64 ± 4.97	33.12 ± 4.72 *	56.4 ± 10.8	56.4 ± 10.8
				Trans-PRK MMC(−)				123 ± 21, N/S	−3.28 ± 1.22, N/S	2830 ± 302	N/S	29.68 ± 5.28	30.23 ± 5.16 *	58.8 ± 9.6	58.8 ± 9.61

SD: standard deviation; Hz: hertz; SE: spherical equivalent; ECD: endothelial cell density; CV: coefficient of variation; HEX: percentage of hexagonal cells; pre-op: preoperative; post-op: postoperative; PRK: photorefractive keratectomy; SM: specular microscopy; A: measured in central zone; B: measured in paracentral zone; C: measured in peripheral zone; Trans-PRK: transepithelial photorefractive keratectomy; IVCM: in vivo confocal microscopy; MMC: mitomycin C; Epi-LASIK: epithelial laser assisted in situ keratomileusis; LASEK: laser epithelial keratomileusis; N/S: not specified; *: statistically significant difference.

PRK or LASEK treatment with MMC application had no statistically significant impact on ECD, CV, or HEX in two out of five studies [22,32], although a decrease in ECD was observed in the latter [32]. In Nassiri et al. [35] and Adib-Moghaddam et al. [23], the mean loss of ECD and CV was significantly higher in the MMC+ group than in MMC-group. Longer MMC application time and the male sex were independently associated with a greater ECD loss [35] Gharaee et al. [36] noted a significant improvement in CV 6 months after PRK with refractive error-graded MMC application. CV change was not related to the duration of MMC application during the surgery.

ECD, CV, and HEX changes were unrelated to ablation depth [22,29,37,38,42,45], the residual stromal thickness [22,29], the total number of pulses delivered [38], the total amount of energy used [38], IOP increase [38], and the duration of follow-up [45].

### 3.2. LASIK and FemtoLASIK

One study out of seven included articles explored corneal endothelial changes following LASIK, one study analyzed FemtoLASIK, and five studies also compared postoperative endothelial parameters between LASIK and FemtoLASIK groups (Klingler et al. [33]), LASIK and PRK groups (Pallikaris et al., 1994 [28], Patel et al. [29], Amoozadeh et al. [30]), or FemtoLASIK and SMILE groups (Shaaban et al. [34]). Two studies explored flap procedures in contact lens (CL) wearers and non-contact lens (non-CL) wearers (Munoz et al. [25], Klingler et al. [33]). Characteristics and results of LASIK and FemtoLASIK studies are presented in Table 2.

No significant differences in ECD, CV, and HEX between the preoperative and postoperative values were reported in three out of seven analyzed studies (Simaroj et al. [24], Amoozadeh et al. [30] Klingler et al. [33]). One author observed a decrease in both ECD and CV, along with a simultaneous increase in HEX (Patel et al. [29]). In turn, a decrease in both ECD (by −1.55%) and HEX (by −5.05%), along with a simultaneous increase in CV was noted in a study by Shaaban et al. [34] at 6 months after FemtoLASIK. A significant decrease in ECD following microkeratome LASIK was also noted by Pallikaris et al. (1994) [28]. They found that the mean percentage of ECD loss at 6 months after LASIK was 6.49%, which increased to 8.67% at 12 months. In another study, ECD was significantly decreased by 6.3% at 9 years after LASIK (Patel et al. [29]). In a study by Munoz et al. [25], both mean ECD and mean HEX increased significantly 12 months after FemtoLASIK compared to preoperative data in the contact lens group, whereas no statistically significant changes in the mean ECD nor HEX were observed in the non-contact lens group. The mean ECD increased by an average of 5.5% in CL-wearers following FemtoLASIK. ECD, CV, and HEX changes following flap procedures were not related to either the AD or RST (residual stromal thickness) [29].

### 3.3. Femtosecond Lenticule Extraction

Two out of three included studies explored corneal endothelial changes following SMILE (Kamiya et al. [26], Zhang et al. [27]) and one study compared postoperative endothelial parameters between SMILE and FemtoLASIK groups (Shaaban et al. [34]). No significant differences in ECD, CV, and HEX occurred between the preoperative and postoperative values in two out of three analyzed studies (Kamiya et al. [26], Zhang et al. [27]), although a mean loss of ECD of 1.7% was observed by Kamiya et al. [26]. A statistically significant decrease in both ECD (by −4.11%) and HEX (by −8.05%), along with a simultaneous increase in CV was only noted in a study by Shaban et al. [34] at 6 months after SMILE. No significant correlations were found between the changes in ECD, CV, or HEX and the degree of SE correction [26] or the estimated RST [27]. Characteristics and results of femtosecond lenticule extraction studies are presented in Table 3. 

### 3.4. Comparative Studies

Three authors evaluated corneal endothelial changes after PRK and LASIK (Pallikaris et al. (1994) [28], Patel et al. [29], Amoozadeh et al. [30]). In the first study, the trial investigators reported that the percentage of endothelial cell loss at 6 months was 6.49% in the LASIK group and 5.69% in the PRK group, which increased to 8.67% and 10.56%, respectively, at 12 months after the refractive procedure. In the second trial [29], ECD was significantly decreased by 6.3% in the LASIK group at 9 years after surgery, whereas in the PRK group, the ECD difference was not statistically significant. Eyes that underwent PRK, CV, and HEX improved significantly over a nine-year period. Finally, the authors of the third study [30] reported that, postoperatively, endothelial cell count increased insignificantly by 0.27% and 1.39% in the LASIK and PRK group, respectively. The HEX increased insignificantly both after LASIK and PRK, but no differences were observed between study groups.

Diakonis et al. [32], who evaluated alterations in ECD after PRK with MMC and Epi-LASIK without MMC, showed that both study groups achieved similar postoperative ECD values. In eyes that underwent PRK or PRK with MMC or LASEK, ECD did not change significantly over 12 months in any treatment group [31].

Klingler et al. [33], who assessed five-year corneal endothelial changes in eyes that underwent LASIK and FemtoLASIK, reported that ECD, HEX, and CV did not differ between the two treatments at any time point. At 5 years, the mean endothelial cell loss was −0.8 ± 5.8% for FS laser treatments and −0.4 ± 5.0% for mechanical microkeratome surgery. Additionally, the authors found no differences in ECD, CV, or HEX between contact lens wearers and non-contact lens wearers both before surgery and 5 years postoperatively.

Shaaban et al. [34] compared the effects of FemtoLASIK and SMILE on corneal endothelial cell parameters over a six-month follow-up. No difference was found between ECD values after one month in the FemtoLASIK group or after three months in the SMILE group. At 6 months, the mean ECD change was −1.55 ± 1.23% in the FemtoLASIK group and −4.11 ± 2.77% in the SMILE group. CV significantly increased in both groups throughout the study. HEX significantly decreased in both groups throughout the study. The mean HEX change was −3.86 ± 2.20% in the FemtoLASIK group and −7.04 ± 2.17% in the SMILE group at 1 month, which further increased to −5.05 ± 1.99% and −8.05 ± 2.00%, respectively, at 6 months postoperatively.

## 4. Discussion

The effects of specific laser refractive techniques on the corneal endothelium are still under research. Some early experimental studies have revealed quantitative and qualitative changes in the corneal endothelium following LRS [46,47,48]. To the best of our knowledge, based on a thorough review of publications, this is the first systematic review of prospective studies assessing the impact of different corneal surface and stromal refractive procedures on the human corneal endothelium. The impact of the following methods was analyzed: PRK with or without MMC, LASEK, Trans-PRK, LASIK, FemtoLASIK, and SMILE.

In this review, both SM and IVCM were used for corneal endothelium analysis. Specular microscopes project a magnified reflection of light on the corneal endothelium. Both non-contact and contact devices are available. Conversely, confocal microscopy employs focused illumination through a spatial pinhole to selectively isolate different layers of the analyzed tissue [48]. Various quantitative techniques of SM may be employed to gather information about the corneal endothelium. The frame method involves quantifying ECD by counting the cells within a specific frame. In the corner method, the intersecting sides of the endothelial image frame are located. The center method identifies the central point of the endothelial cell and then locates cells situated nearby. Conversely, the comparison method provides a subjective assessment of ECD by visually comparing the image to known hexagonal patterns for various cell densities. Most specular microscopy devices automatically detect the boundaries of endothelial cells for cell analysis [49].

The precision of scans holds great significance, with key elements influencing repeatability being the proficiency of the photographer and minimal cellular polymegathism, indicative of a clinically normal endothelium. Benetz et al. discovered that assessments conducted by two technicians on 688 images matched only 64% of the time [50]. For the highest reliability and repeatability in the subjective identification of cells, it is recommended that a single technician analyzes all the specular micrographs [11]. In vivo confocal microscopy provides high magnification and resolution of corneal structures, even when corneal opacity or edema co-exists. It can capture both the central and peripheral corneal regions [12]. An evaluation comparing noncontact specular microscopy (Noncon Robo, Konan Medical, Irvine, CA, USA) and confocal microscopy (ConfoScan 3, Nidek Technologies, Albignasego PD, Italy) revealed no disparity in ECD. Nevertheless, the agreement between the devices relied on the specific data analysis method employed [51].

Imre et al. conducted a study on IVCM and determined that the automated analysis of endothelial data exhibited reliability and reproducibility. In the manual evaluation, any interobserver differences noted were considered clinically insignificant. The authors concluded that the variances between automatic and manual analysis methods might be attributed to technical aspects related to the measurement [52].

Several factors need to be considered in terms of potential endothelial alterations following LRS in myopic eyes: firstly, the direct damage due to laser radiation exposure; secondly, the immediate cell damage caused by the acoustic and resonance waves produced by the laser pulse on the cornea; thirdly, corneal metabolic changes due to the absence of the Bowman’s membrane and the thinning of the cornea; fourthly, modifications in corneal metabolism associated with the cessation of contact lens use before surgery; fifthly, the toxicity of MMC; and sixthly, steroid-related postoperative IOP increases.

When assessing the impact of LRS on the corneal endothelium, we decided to include research data with at least a six-month follow-up for two reasons: firstly, to obtain a more complete assessment of the corneal endothelium biology that might be missed in a three-month follow-up, and secondly, to reduce the potential impact of short-term fluctuations in endothelial parameters related to corneal healing processes in the early postoperative period. Some articles included in this analysis provided several checkpoint assessments to capture both short-term and long-term effects.

### 4.1. The Impact of the Excimer and/or Femtosecond Lasers

Both excimer and femtosecond lasers may, in theory, be regarded as potentially harmful due to the energy emitted during treatment. Possible mechanisms include mechanical trauma from shock waves, thermal effects, secondary ultraviolet radiation, and metabolic changes [13,14].

The ultraviolet light with a wavelength of 193 nm emitted by the excimer laser during ablation appears to be completely absorbed at the point of impact and penetrates less than the diameter of a cell [53]. In turn, the femtosecond (FS) laser is a type of neodymium laser with a wavelength in the near infrared range (1040–1053 nm). It can focus with very short pulses and create incisions by producing cavitation bubbles and a split interface during the photodestruction of the corneal stroma [54].

During three decades of dynamic development of laser technology, the parameters, including frequencies, energy, and safety profiles, have changed significantly. The initial versions of excimer lasers were “broad beam” lasers which might produce heat dissipation effects. The recent “flying spot” excimer laser systems, featuring small spot sizes, very high pulse frequency, and very quick and precise spot distribution, may help to avoid thermal effects [55].

Also, femtosecond laser technology has been significantly improved since its introduction in 2001. The latest femtosecond laser generation can achieve a pulse rate of 2 MHz, which enables the preparation of the corneal flap in only a few seconds. Moreover, a higher pulse rate in combination with lower total energy delivered to the cornea and lower vacuum pressure needed to stabilize the eyeball contribute to the much higher safety profile of the femtosecond laser LASIK procedure as compared to the mechanical keratome LASIK method [56].

An experimental study conducted on rabbit eyes revealed that excimer laser ablation of the stroma within 200 microns of the corneal endothelium results in structural changes in the endothelial cells and the formation of amorphous (granular material) deposits in Descemet’s membrane [57] It was demonstrated by Kim et al. [58] that LASIK surgery might produce acute morphological changes in the corneal endothelium that regress rapidly. Significant alterations in the shape and appearance of cells were observed, characterized by distortions in the hexagonal shape and scattered regions of cell darkening indicative of endothelial cell edema. These modifications were widespread across the specular microscopy image but reverted to the normal status by the first day after surgery. The location and central distribution of the endothelial abnormalities suggested an adverse response to excimer laser ablation and/or the direct effect of increased IOP generated by suction. The changes in endothelial cell parameters were similar in all 20 eyes immediately after the procedure and did not correlate with ablation depth (AD) or RST.

The hypothesis of a propagating acoustic wave effect seems to be confirmed by Trocme et al. [37], who found a statistically significant decrease in peripheral ECD in eyes after PRK during a 12-month postoperative period, with little effect on the central endothelium. A 193 nm excimer laser with a frequency of 10 Hz was applied in this study, and the pulse energy was 180 mJ/cm^2^. Pallikaris et al. (1994) [28] observed a statistically significant reduction in ECD following PRK and LASIK procedures, utilizing an excimer laser with an energy of 220 mJ/cm^2^ and a repetition rate of 20 Hz. Mardelli et al. (1995) [45] reported a decrease in both CV and HEX after PRK, employing an energy of 160 mJ/cm^2^ and a repetition rate of 5 Hz. Carones et al., 1994 [38] documented a decrease in CV and an increase in HEX after PRK, using a pulse energy of 180 mJ/cm^2^ and a repetition rate of 10 Hz.

Mardelli et al. [45] examined the long-term effects of PRK on the corneal endothelium. PRK caused no damage to the central corneal endothelium. According to the authors, polymegathism differences in the treated and untreated eyes could be attributed to modifications in corneal metabolism or to a yet unknown mechanism. The 193 nm excimer laser beam was delivered with a 5 Hz repetition rate with the fluence adjusted to 160 mJ/cm^2^. Each pulse of this laser ablated approximately 0.3 μm of the anterior stroma. Simaroj et al. [24] examined the corneal endothelium in patients who underwent LASIK myopic correction. No significant changes were observed in ECD or CV, although an average ECD loss of 1.54% was noted during a twelve-month observation. Ablations were performed with an excimer laser with a repetition rate of 30 Hz and the energy fluence applied ranging from 90 to 100 mJ/cm^2^. Conversely, Pallikaris et al. [28] compared LASIK and PRK regarding their potential influence on the corneal endothelium in myopic patients, and both methods resulted in a statistically significant loss of endothelial cells during 12 months of follow-up time. Interestingly, 6 months after refractive surgery, the loss was greater (6.84%) in the LASIK group than in the PRK group (5.69%). After 12 months of follow-up, the trend reversed, and endothelial cell loss was greater in PRK (10.56%) than in LASIK (8.67%). Ablations were performed with the same device with identical settings for both PRK and LASIK. The 193 nm excimer laser with a firing rate of 20 Hz and energy fluence set at 220 mJ/cm^2^ was used. To conclude, the authors considered those two techniques as generally safe for the corneal endothelium. They viewed the obtained results as acceptable and believed the cell loss in both methods was caused by the rearrangement of central endothelial cells. In this study, the morphology of the cells was not assessed.

In theory, as regards eyes that have undergone FemtoLASIK, the corneal endothelium may be more significantly affected compared to cases of excimer laser ablation alone, as additional shock waves produced by the FS laser during flap preparation, combined with the energy from the excimer laser during ablation, could contribute to a greater impact on the corneal endothelium [15,16]. However, Klingler et al. [33] did not demonstrate a difference in ECD between fellow eyes within a five-year follow-up where one eye underwent LASIK with a flap created with a microkeratome and the other one with an FS laser. The ECD values did not differ from the preoperative data in either treatment group in this long-term study.

Nevertheless, Netto et al. [17] conducted an animal model study and showed that refractive surgery could negatively impact the cornea through various mechanical, photodestructive, and photochemical mechanisms. The authors investigated stromal wound healing and inflammation after FS and microkeratome flap creation. LASIK flaps were generated using three different femtosecond laser models operating at 15 KHz, 30 KHz, and 60 KHz. Both Muñoz et al. [25] and Klinger et al. [33] used a 15 kHz FS laser to create flaps, but the results obtained in their studies did not stay in line with Netto’s findings [17]. Netto et al. [17] concluded, that stromal cell necrosis associated with FS laser flap formation likely contributed to greater inflammation and greater keratocyte death as compared to microkeratome LASIK. In another study, Helena et al. [59] demonstrated that the microkeratome-induced flap formation was mostly linked to keratocyte apoptosis, whereas the FS laser caused energy-related cell necrosis. They found that necrosis was a more significant stimulus to inflammatory cell infiltration than apoptotic bodies. However, they concluded that necrotic debris should not negatively influence the cellular recovery of the cornea or the clinical outcomes of LASIK performed with the FS laser as long as the inflammation is properly managed with topical corticosteroids [17].

Inflammatory pathways and arachidonic acid metabolites in the cornea may theoretically trigger inflammation in the anterior chamber. This was not confirmed by Özbilen et al. [60] in a three-month FemtoLASIK study. At the same time, they found a statistically significant 3.3% decrease in ECD.

In the SMILE procedure, an FS laser is only used for the creation of a refractive lenticule, which is then manually extracted through a small 2–3 mm incision. Shaaban et al. [34] compared the effect of FemtoLASIK and SMILE on corneal endothelium cells.

No details regarding the FS laser energy/repetition rate or the flap thickness or cap were provided in the study. They reported statistically significant changes in ECD, CV, and HEX in both study groups at 6 months postoperatively as compared to the preoperative values. However, the SMILE group was more affected. At 6 months, the respective mean ECD and HEX changes were −1.55 ± 1.23% and −5.05 ± 1.99% in the FemtoLASIK group and −4.11 ± 2.77% and −8.05 ± 2.00% in the SMILE group. The fact that the SMILE group was more affected may be due to the deeper penetration of both laser energy and generated shock waves into the corneal tissue. However, Kamiya [26] and Zhang [27] observed no significant changes in corneal endothelial parameters after SMILE was performed with a 500 kHz femtosecond laser.

### 4.2. The Role of Attempted Correction (ES, AD) and Residual Stromal Thickness (RST)

Special attention should be paid to myopes qualified for LVC due to endothelial morphological changes and the loss of barrier function that may occur after deep stromal ablation. RST above 250 µm [61] is strongly recommended. The SMILE criteria also require postoperative stromal bed thickness to be at least 250 microns [62].

Ten studies included in this review examined the impact of SE, AD, and RST on endothelial parameters. Pallikaris et al. [28] revealed a reduction in central corneal endothelial cell density at 6 months, with a smaller decrease observed at 24 months post-LASIK in eyes with moderate to high myopia. The extent of endothelial cell loss was more significant in patients who underwent higher attempted corrections. None of the remaining nine studies mentioned above ([22,26,27,29,33,37,38,42,45]) revealed any significant correlations between endothelial parameters (such as ECD, CV, HEX) and preoperative SE, AD, or RST.

Mohammad-Rabei et al. [63] (not included in this review) indirectly investigated the impact of AD (≥65 µm or <65 µm) on the corneal endothelium after PRK or LASEK with or without mitomycin (MMC) in 171 patients up to 6 months postoperatively. The obtained results suggested that larger AD, coupled with the use of MMC, might be associated with decreased hexagonality and increased CV in the corneal endothelium following those procedures.

### 4.3. The Effect of Contact Lenses (CL)

Some authors demonstrated an increase in ECD after LASIK performed with a microkeratome among contact lens wearers [64,65]. The results were in accordance with the data obtained by Muñoz et al. [25], who noted a statistically significant increase in ECD in the eyes of contact lens wearers after 15 kHz femtosecond LASIK.

Carones et al. [38] found significant changes in CV and HEX at 12 months after PRK. The cessation of contact lens use led to a reorganization of the endothelial structure with positive changes in polymegathism and pleomorphism. All the patients with better indices after PRK were contact lens users.

Conversely, Trocme et al. [37] found a statistically significant decrease in peripheral ECD in myopic eyes of contact lens wearers who underwent PRK. The authors observed a significant difference in ECD between the central and peripheral zones of the cornea preoperatively, and they suggested that such a disparity might have been caused by the chronic use of contact lenses, which may lead to the redistribution of endothelial cells with higher cell density in the periphery. No relationship between endothelial parameters and contact lens use following LASIK and FemtoLASIK was found by Klingler et al. [33] in a five-year study.

The cessation of contact lens use is considered a key mechanism responsible for endothelial cell density improvement, although this process seems to be more complex. Wiffen et al. [66] compared peripheral and central endothelium in both normal patients and long-term contact lens wearers. They suggested that contact lens use caused a slight redistribution of endothelial cells from the central to the peripheral cornea and that the reversal of this redistribution could mask slight central endothelial damage resulting from refractive surgery. Collins et al. [53] conducted a retrospective study which showed no significant alteration of ECD or HEX after a three-year follow-up in patients who underwent LASIK with a microkeratome. Both mechanical and femtosecond methods caused no significant impact on the endothelial cell density of myopic patients who had been non-contact lens wearers preoperatively [64]. Some authors suggested that the modification of corneal metabolism due to the absence of the Bowman’s membrane and the thinning of the stroma might be another mechanism involved. This may lead to a greater oxygen flow through the cornea from its outermost layers towards deeper layers [67]. Furthermore, the results obtained in a long-term study by Patel et al. [29] supported the hypothesis that LASIK and PRK were safe for the corneal endothelium. According to the authors, the average endothelial cell loss after both methods was similar to age-related annual cell depletion in healthy patients. When analyzed together, no significant changes in endothelial cell morphology were found in eyes that underwent LASIK or PRK. However, an improvement occurred in the CV and HEX in subjects who underwent PRK. While the cessation of contact lens use may have contributed to this fact, the authors expected a similar improvement in the eyes that underwent LASIK, since the majority of those eyes had also used contact lenses before surgery. Notably, neither the amount of ablated tissue nor the thickness of the residual bed were associated with endothelial cell loss. For both LASIK and PRK, the stroma was ablated with a VISX Star excimer laser. No detailed information regarding laser energy or frequency was provided by the authors. Interestingly, the authors postulated that post-LASIK and PRK corneas could be considered suitable for posterior lamellar keratoplasty due to the lack of difference in endothelial cell loss in comparison with normal patients.

### 4.4. The Role of Mitomycin (MMC)

MMC is an antibiotic isolated from cultures of *Streptomyces caespitosus*. The drug acts as an alkylating agent that inhibits DNA synthesis and, as a consequence, cell mitosis. In eyes undergoing surface ablation, MMC reduces the incidence and severity of postoperative corneal haze by inhibiting fibroblastic proliferation in the ablated stroma [68].

Torres et al. [69] found a measurable concentration of MMC in the anterior chamber after the topical application to the cornea, suggesting that the active molecules came into contact with the deep stroma and the endothelium, with a potentially toxic effect.

De Benito-Llopis et al. [70] found that MMC helped prevent the development of clinically relevant haze during surface ablation. In addition, an intraoperative application of MMC 0.02% for 30 s caused no significant change in ECD. A recent study by Al-Mohaimeed et al. [71] also revealed the safety and efficacy of the use of MMC in reducing corneal haze after Trans-PRK in low to high order myopia. MMC exerted no effect on the number or density of corneal endothelial cells.

Conversely, Adib-Moghaddam et al. [23], whose study is included in this analysis, demonstrated that cell density loss was significantly higher in the MMC-treated right eyes of patients compared to their left eyes with no MMC application. CV was also significantly higher in the right eyes. According to the authors, all ablation procedures were performed with a Schwind Amaris 500 Hz excimer laser with its integrated CAM software. The authors provided no details concerning the laser energy applied. These results may be partly explained by the fact that ablation depth in Trans-PRK is the sum of epithelial and stromal ablations. It is expectedly higher than PRK for the comparable degree of myopia, as the latter method only involves stromal ablation.

Moreover, Nassiri et al. [35] found that ECD was significantly more reduced after PRK in a group with MMC 0.02% (0.2 mg/mL) than in a group without MMC (−14.8% vs. −5.1%). The length of application depended on the ablation depth and varied from 10 up to 50 s. Despite the fact that endothelial cell loss was the greatest during the first postoperative week, the depletion gradually continued up to the sixth month. The authors presumed that this might be due to the prolonged activity of MMC and concomitant free radicals. Interestingly, extended MMC exposure time as well as the male sex were the only independent risk factors for greater endothelial cell loss. No detailed information regarding laser energy or frequency was provided by the authors.

The authors recommended the use of MMC at minimal therapeutic concentrations for the shortest effective duration. Neither the age, depth of ablation, nor postoperative corneal thickness were independently associated with endothelial cell loss after surgery.

Gharaee et al. [36] reported that CV was the only parameter that was significantly elevated (*p* = 0.016) with no change in ECD or cell size in a six-month follow-up. A flying spot excimer laser with an emission wavelength of 193 nm, a fixed pulse repetition rate of 100 Hz, and a radiant exposure of 400 mJ was used in this study.

The dose and duration of MMC, the follow-up periods, the PRK method, and the ethnic descent of the patients may be responsible for unequivocal results obtained by some researchers. Both the dose and the duration of MMC exposure were considered important correlates of cytotoxicity in rabbit eyes [19]. MMC exerts its effect by binding strands of the DNA double helix, thus affecting the corneal endothelium and possibly leading to corneal decompensation. According to Majmudar et al. [72], MMC use in low myopia is not always necessary. However, the need for MMC use in moderate and high myopia is considered reasonable.

Despite the fact that most clinical studies supported the statement that 0.02% MMC used for up to 2 min was safe for the corneal endothelium, MMC should be used with caution, especially among patients with low myopia.

### 4.5. Laser Refractive Surgery in Corneal Dystrophies

Corneal dystrophies are generally regarded as relative or absolute contraindications to laser keratorefractive surgery as they may significantly contribute the safety profile and refractive outcomes of the procedures [73]. Only one case-report with Fuchs endothelial corneal dystrophy (FECD) [20] and one retrospective case series of seven eyes with corneal guttata undergoing LASIK [74] were published. Vroman et al. reported a single case of a 58-year-old woman with preoperative corneal guttata in the right eye and mild Fuchs endothelial corneal dystrophy (FECD) and concomitant trace corneal edema in the left eye who underwent bilateral hyperopic LASlK. The preoperative central pachymetry was 585 μm in the right eye and 656 μm in the left eye. No information regarding ECD measurement performed preoperatively was provided by the authors. Seven months after LASIK treatment, the patient developed transient postoperative corneal edema in the right eye but persistent decompensation of the cornea in the left eye, with a central pachymetry of 607 μm in the right eye and 782 μm in the left eye. Postoperatively, visual acuity decreased from 20/20 preoperatively to 20/40 in the right eye and from 20/20 to 20/400 in the left eye. Finally, penetrating keratoplasty was performed in the left eye 14 months after LASIK. The histopathological analysis of the cornea revealed a significant degeneration of the endothelial cell layer with nuclear pyknosis and the disappearance of the cellular organelles. Also, significant depositions of mucopolysaccharide components along the interface between the flap and the residual stroma were found. Regarding refractive outcomes and endothelial changes in myopic eyes with corneal guttata undergoing uneventful LASIK, Moshifar et al. reported a loss of two lines in corrected distance visual acuity (CDVA) in six out of seven eyes (86%), myopic shift, a 12.4% decrease in ECD, and an increase in CCT one year after surgery. Three studies reported positive and safe one-year results of LASIK (four eyes) [75], PRK (fourteen eyes) [76], and SMILE (one eye) [77] procedures in eyes with posterior polymorphous corneal dystrophy (PPCD).

According to the systematic review, unfavourable LASIK and/or PRK results were also found in some anterior (Cogan, Meesmann) and stromal corneal dystrophies (lattice LCD-1, Avellino). To date, insufficient scientific evidence has prevented the recommendation of any refractive surgery procedures in other corneal dystrophies or femtosecond lenticule extraction in any corneal dystrophy [73].

### 4.6. Study Limitations

Several limitations should be considered to ensure the validity and generalizability of findings analyzed in this article. Here are possible limitations specific to the reviewed matter:

1. Heterogeneity in Refractive Surgical Procedures and Equipment: The impact on corneal endothelial cell density may differ depending on the specific refractive surgery techniques, the lasers used, and the range of parameters involved.
2. Diversity in Study Populations: Variability in patient demographics, including the ethnic origin, age, sex, and pre-existing ocular conditions, may occur across studies, potentially influencing endothelial cell counts.
3. Inconsistencies in Measurement Techniques: Differences in the methods used for corneal endothelial cell counting, such as variations in microscopy techniques (specular vs. in vivo confocal) or imaging devices, may introduce measurement bias and affect the reliability of pooled results.
4. Limited Standardization of Reporting: Lack of standardized reporting guidelines for corneal endothelial cell counting after refractive surgery (different techniques) may lead to inconsistent data presentation and hinder the comparability of studies.
5. Patient Selection Bias: Studies may not adequately account for patient selection biases, such as excluding individuals with certain risk factors or comorbidities, potentially limiting the generalizability of the findings.
6. Small Study Effects: Small studies with limited sample sizes may show larger effects than larger studies, leading to potential bias when pooling the results.

## 5. Conclusions

- The corneal endothelium is not directly affected by the LRS. However, the energy of the laser, pressure changes, and surgical manipulations during the procedure may lead to minor and transient endothelial cell loss. This loss is usually well within the cornea’s regenerative capacity.
- While short-term studies often show minimal (if any) impact on the corneal endothelium, the long-term effects of laser vision correction are still being studied. Most research indicates that the endothelium remains stable over time for the vast majority of patients. Nevertheless, individual factors and variations in LRS methods may play a role.
- Patient selection is critical to ensuring safety. Individuals with pre-existing corneal conditions or endothelial issues (Fuchs’ dystrophy) may not be good candidates for laser vision correction.
- In general, most studies indicate that PRK, LASEK, LASIK, FemtoLASIK, and SMILE procedures do not significantly affect the corneal endothelium. However, some studies revealed that laser refractive treatment might cause endothelial cell density and morphological changes. Future studies are needed to further elucidate the possible effect of excimer and femtosecond laser procedures on the corneal endothelium.

## Figures and Tables

**Table 2:** Characteristics and results of LASIK and FEMTOLASIK studies.

Study	Study Size		Age (Mean, SD, Range) (Years)	Correction Method	Excimer Laser Firing Frequency (Hz)	Femtosecond Laser Firing Frequency (kHz)	Exam Method	Follow-Up Time (Mean, SD, Range) (Months)	Ablation Depth (Mean, SD, Range) (µm)	SE (Mean, SD) (Diopters)	ECD (Mean, SD)		CV (Mean, SD)		HEX (Mean, SD)	
	Patients	Eyes								Pre-Op	Pre-Op	Post-Op	Pre-Op	Post-Op	Pre-Op	Post-Op
Pallikaris I.G. et al., 1994 [28]	20	10	N/S, N/S, (31–58)	LASIK	20	Microkeratome	SM	12, N/S, N/S	N/S	−11.00 ± 0.86 (−8.00–−16.00)	2744 ± 217	2644 ± 235 *	N/S	N/S	N/S	N/S
Simaroj P. et al., 2003 [24]	105	180	32 ± 9 (16–55)	LASIK	30	Microkeratome	SM	12, N/S, N/S	N/S	N/S, N/S, (−0.75–−15.00)	2547 ± 282	2508 ± 290	58.33 ± 11.50%	58.61 ± 9.89%	N/S	N/S
Patel S. et al., 2009 [29]	16	20	34 ± 8 (23–47)	LASIK	N/S	Microkeratome	SM	108, N/S, N/S	66 ± 34, N/S	−6.20 ± 1.40 (−4.00–−9.25)	2925 ± 303	2741 ± 308 *	0.33 ± 0.05	0.33 ± 0.03	55.0 ± 9.0	56.0 ± 5.0
Amoozadeh J. et al., 2009 [30]	4	8	28 ± 7 (19–36)	LASIK	N/S	Microkeratome	IVCM	6 N/S, N/S	61 ± 17 (36–71)	−2.94 ± 0.96 (−2.00–−4.25)	3022 ± 224	3030 ± 186	N/S	N/S	52.2 ± 11.4	53.0 ± 7.6
Muñoz G. et al., 2011 [25]	62	62	32 ± 7 (21–42)	FemtoLASIK CL(−)	N/S	15	SM	12 N/S, N/S	45 ± 30 (12–123)	N/S, N/S, (−0.75–−9.00)	3605 ± 219	3564 ± 232	N/S	N/S	35.1 ± 11.7	34.4 ± 11.2
	76	76		FemtoLASIK CL(+)	N/S	15			48 ± 23 (19–113)	N/S, N/S, (−0.75–−9.00)	3401 ± 292	3587 ± 262 *	N/S	N/S	31.0 ± 5.1	32.5 ± 4.0
Klingler K. et al., 2012 [33]	18	17	38 ± 10 (22–54)	FemtoLASIK	N/S	15	IVCM	60, N/S, N/S	N/S	N/S	2758 ± 406	2829 ± 317	32.00 ± 6.00	31.00 ± 4.00	59.0 ± 8.0	57.0 ± 7.0
		17		LASIK	N/S	Microkeratome					2773 ± 399	2853 ± 355	32.00 ± 6.00	32.00 ± 3.00	59.0 ± 6.0	57.0 ± 6.0
Shaaban Y.M. et al., 2020 [34]	20	40	29 ± 5 (19–37)	FemtoLASIK	N/S	Microkeratome	SM	6, N/S, N/S	N/S	−4.06 ± 1.94, N/S	2947 ± 151	2901 ± 150 *	29.48 ± 2.87	31.75 ± 3.27 *	60.1 ± 2.9	57.1 ± 3.2 *

SD: standard deviation; Hz: hertz; kHz: kilohertz; SE: spherical equivalent; ECD: endothelial cell density; CV: coefficient of variation; HEX: percentage of hexagonal cells; pre-op: preoperative; post-op: postoperative; SM: specular microscopy; IVCM: in vivo confocal microscopy; CL: contact lens; N/S: not specified; *: statistically significant difference.

**Table 3:** Characteristics and results of femtosecond lenticule extraction studies.

Study	Study Size		Age (Mean, SD, Range) (Years)	Correction Method	Femtosecond Laser Firing Frequency (kHz)	Exam Method	Follow-Up Time (Mean, SD, Range) (Months)	Ablation Depth (Mean, SD, Range) (µm)	SE (Mean, SD) (Diopters)	ECD (Mean, SD)		CV (Mean, SD)		HEX (Mean, SD)	
	Patients	Eyes							Pre-Op	Pre-Op	Post-Op	Pre-Op	Post-Op	Pre-Op	Post-Op
Kamiya K. et al., 2012 [26]	20	38	33 ± 8 (20–48)	Femtosecond Lenticule Extraction	500	SM	6 N/S, N/S	N/S	−4.26 ± 1.39 N/S, (−2.00–−7.00)	2814 ± 199	2762 ± 213	N/S	N/S	N/S	N/S
Zhang H. et al., 2015 [27]	30	56	26 ± 6 (18–40)	Femtosecond Lenticule Extraction	500	SM	12 N/S, N/S	N/S	−5.50 ± 1.10 (−3.50–−8.25)	2840 ± 322	2837 ± 311	31.90 ± 4.90	32.10 ± 4.60	58.7 ± 9.3	58.5 ± 8.5
Shaaban Y.M. et al., 2020 [34]	20	40	28 ± 5 (19–37)	Femtosecond Lenticule Extraction	N/S	SM	6 N/S, N/S	N/S	−5.18 ± 1.86 N/S	3038 ± 161	2913 ± 170 *	28.38 ± 2.24	33.03 ± 2.47 *	61.7 ± 2.6	56.7 ±2.5 *

SD: standard deviation; kHz: kilohertz; SE: spherical equivalent; ECD: endothelial cell density; CV: coefficient of variation; HEX: percentage of hexagonal cells; pre-op: preoperative; post-op: postoperative; SM: specular microscopy; N/S: not specified; *: statistically significant difference.

## Data Availability

The data presented in this study are available on request from the corresponding author.
